# Effect of Deposition Parameters for Ni-Au Coatings on Corrosion Protection Properties of 2A12 Aluminum Alloy

**DOI:** 10.3390/ma18050969

**Published:** 2025-02-21

**Authors:** Shipeng Chen, Jinglan Xu, Dingchuan Huang, Weiwei Zhang, Tian Zhang, Liangliang Xiong, Xiaoqiang Fan

**Affiliations:** 1 Institute of Electronic Engineering, China Academy of Engineering Physics, Mianyang 621999, China; chensp_hit@163.com (S.C.);; 2 Key Laboratory of Advanced Technologies of Materials (Ministry of Education), School of Materials Science and Engineering, Southwest Jiaotong University, Chengdu 610031, China

**Keywords:** aluminum alloy, electrodeposition coating, anti-corrosion, grey theory

## Abstract

The Ni-Au coating with its inherent chemical stability is recognized as an effective method for boosting corrosion resistance in humid environments while preserving exceptional electrical conductivity. However, its anti-corrosion performance is affected by the structure characteristics of the coating due to the high corrosion potentials of Au and Ni. To enhance its protection properties, the deposition process parameters, including deposition time, deposition current density, and zincating times, were investigated. The morphology and structure of the coatings were characterized, while its anti-corrosion performance was assessed through electrochemical and accelerated salt-spray tests. Eventually, the elevated current density in the Ni-Au coating resulted in reduced grain size and improved surface morphology, ensuring superior anti-corrosion performance. Additionally, extending the Ni deposition time provided a second physical barrier for the dense and thick Ni layer to resist the invasion of corrosive media. Furthermore, grey theory was applied to predict the service life of the Ni-Au coating. This research provides valuable insights and constructive guidance for optimizing Ni-Au coating in various engineering applications.

## 1. Introduction

2A12 aluminum alloy is widely used in aerospace shells, brackets, and rotating deployment mechanisms due to its advantages of low manufacturing cost, high specific strength, low density, and easy mechanical processing [1,2,3,4]. However, with the rapid advancements in national economic and defense industry construction, traditional 2A12 Al alloy materials are increasingly unable to meet the demands posed by use in severely corrosive environments due to susceptibility to pitting [2,5]. Certain second phases, particularly Cu-containing phases such as *θ* phase (Al_2_Cu) and *S* phase (Al_2_CuMg), which commonly manifest as larger particles, impede the formation of passivation coatings on the surface of Al alloys, resulting in a discontinuous and defective passivation coating [6,7,8,9,10].

To mitigate or impede the corrosion behavior of aluminum alloys and enhance their service life in humid environments, appropriate corrosion protection treatments must be implemented on the aluminum alloy surface. Currently, the protection methods for 2A12 aluminum alloy can be divided into four categories: corrosion inhibitors [11,12,13,14], surface oxidation treatments [15,16,17,18,19], and metal or organic protective coatings [13,20,21]. Organic coatings possess favorable mechanical properties and constitute an economical approach to corrosion protection [22,23,24]. By contrast, metal coatings offer superior electrical and thermal conductivities, rendering them suitable for applications necessitating electrical connections or heat dissipation. Generally, a metal coating is prepared primarily by electroplating or chemical plating. Directly depositing the target coating onto Al alloy results in poor uniformity and weak interface adhesion. To overcome this, a pre-Zn and Ni plating step is necessary prior to electroplating, followed by the application of target coatings onto the Ni layer to ensure protection [25].

Extensive research has shown that the synergistic effect of Au with its inherent chemical inertness and a Ni layer with enhanced bonding strength provided significant resistance to the penetration of corrosive media in harsh environments [26]. Kong et al. [27] studied the corrosion resistance mechanism of the porosity and thickness of the Ni-Au coating under a corrosive atmosphere. Lee et al. [28] evaluated the impact of both thickness and porosity of the Ni-Au coating on its anti-corrosion performance by employing an accelerated corrosion testing methodology. They collectively contributed to an enhanced comprehension of the influence exerted by the inherent characteristics of the Ni-Au coating on its anti-corrosion performance. However, it is crucial to note that various process parameters play a pivotal role in determining the structural properties and corrosion resistance of the coating, which deserves for further exploration.

In this work, a comprehensive and systematic investigation was conducted to examine the effects of various processing parameters, including deposition time, deposition current density, and times of Zn immersion, on both the morphological and structural features of the Ni-Au coating formed on the 2A12 Al alloy. Furthermore, the anti-corrosion performance was analyzed through electrochemical and salt spray tests. Simultaneously, a grey theory model was performed to elucidate the corrosion behavior and predict the service life of the Ni-Au coating. The results showed that the influence of current density on thickness, surface morphology, and grain size was more significant than that of deposition time. The findings not only enhanced the understanding of the interplay among these process parameters and the structures of the Ni-Au coating, but also provided a theoretical foundation for assessing the reliability and service life in aggressive corrosive environments.

## 2. Materials and Methods

### 2.1. Materials

Nickel sulfate (NiSO_4_), sodium hypophosphite (NaH_2_PO_2_), citric acid (C_6_H_8_O_7_), and potassium gold cyanide (KAu(CN)_4_) were purchased from Shanghai Aladdin Biochemical Technology Co., Ltd. (Shanghai, China). All commercial chemicals and solvents were used without further purification.

### 2.2. Preparation of Ni-Au Coatings

After removing the oxide layer from the 2A12 aluminum alloy through sanding, the surface was thoroughly cleaned multiple times using alcohol followed by acetone in an ultrasonic cleaning process to ensure complete cleanliness. Subsequently, the cleaned 2A12 aluminum alloy was sequentially immersed in the plating solution, undergoing twice the primary zincating process, followed by nickel (Ni) electroplating for 12 min, and finally, pulse gold electroplating was performed at a current density of 0.2 A/dm^2^ for 60 min in a plating solution with a pH of 4.8. This sample was labeled as Al-Au-1. The deposition process parameters were then changed to explore their influence. The comprehensive parameters and detail are provided in Table 1.

### 2.3. Characterization of Ni-Au Coatings

The microscopic morphological analysis of both the surface and cross-section of the Ni-Au coating was performed utilizing field emission scanning electron microscopy (FESEM, JSM-6610, JEOL, Tokyo, Japan). In addition, the elemental composition and distribution of the Ni-Au coating were analyzed using an equipped X-ray energy dispersive spectrometer (EDS, X-Max 80, Oxford, UK). X-ray diffraction (XRD, Bruker D8ADVANCEA25X, Bruker, Singapore) was employed to ascertain the microstructure of the Ni-Au coating and crystalline state. The adhesion strength between the coating and the substrate was evaluated using a scratch tester (MFT-2000, Sky technology Development, Shenyang, China) with a loading rate of 1 N/s, a termination load of 30 N, and a scratch length of 5 mm. The three-dimensional surface morphology before and after corrosion was obtained with a white light interferometer (WLI, Contour GT, Bruker, Billerica, MA, USA). The chemical composition of the coating was analyzed using X-ray photoelectron spectroscopy (XPS, NpFlex, Bruker, USA).

The electrochemical evaluations of all samples were carried out using the Princeton electrochemical workstation with the three-electrode system in 3.5 wt.% NaCl solution. The 2A12 alloy with Ni-Au coating served as the work electrode, accompanied by the saturated calomel electrode (SCE) as the reference and the platinum plate as the counter electrode. Prior to conducting electrochemical impedance spectra (EIS) and potentiodynamic polarization tests, the open circuit potential (OCP) was monitored to ensure potential stability. Following this, EIS was performed across a frequency range of 10^−2^ to 10^5^ Hz with a signal amplitude of 10 mV. Potentiodynamic polarization measurements were carried out by scanning from −0.25 V to 0.25 V (vs. OCP) at a scan rate of 0.5 mV/s. The execution of the salt spray test, an artificially induced accelerated corrosion assessment methodology, was conducted using specialized salt spray testing apparatus, in strict accordance with the guidelines stipulated within the neutral salt spray test standard [29].

### 2.4. Life Prediction of Ni-Au Coatings

The corrosion protection performance of coatings was affected by multiple factors, with the individual contributions to coating degradation being complex and difficult to quantify. Consequently, establishing a functional relationship between coating life and these influencing factors presented a challenging task. To address this issue, grey system theory provided a robust solution. This theory systematically transformed irregular and uncertain factors into quantifiable measures using sophisticated mathematical methodologies. Additionally, electrochemical analysis enabled the characterization of changes in various parameters of the Ni-Au coating during immersion. By integrating the results from salt spray tests, a grey system theory-based life prediction model incorporating electrochemical data is presented in Equation (1) [30].

(1) $$t=\frac{Nln\left[\frac{A\left(1-e^{a}\right)}{lgR_{c}}\right]}{a}+t_{0}$$

where the *t*, *N*, *A*, *a*, *R*_c_, and *t*_0_ represent the anti-corrosion life of the coating, the time interval of the equidistant data, model coefficients, development coefficient, the coating resistance, and the time of initial data, respectively. Among them, *A* is confirmed through $\left[x^{\left(0\right)}(1)-\frac{u}{a}\right]$, where *u* and *a* are confirmed through the least squares method.

## 3. Results and Discussion

### 3.1. Morphologies of Ni-Au Coatings

**Figure 1:**
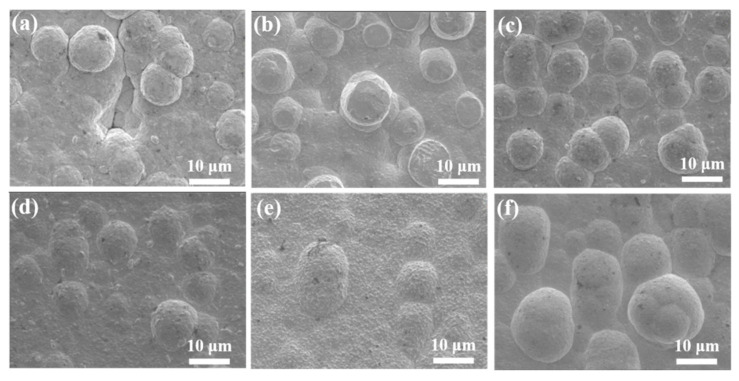
SEM micrographs of (**a**) Al-Au-1, (**b**) Al-Au-2, (**c**) Al-Au-3, (**d**) Al-Au-4, (**e**) Al-Au-5, and (**f**) Al-Au-6.

Figure 1 depicts the surface morphology of the Ni-Au coating on 2A12 aluminum alloy. Notably, all samples exhibited a nodular morphology featuring spherical structures on their surfaces. In Figure 1a, there are pores and microcracks between the nodules, indicating the poor surface conditions of the Al-Au-1. In addition to the nodular morphology, the Al-Au-1, Al-Au-3, and Al-Au-4 samples displayed some small particles with a diameter of 1.4 μm on the alloy surface. This could be related to the electrodeposition parameters of the Au layer, including current density (0.2 A/m^2^) and time (60 min). For the Al-Au-5 and Al-Au-6 samples, the charge deposited per unit area of the Au layer remained constant (Q/S = *i* × *t* = 120 A·min/m^2^), consistent with the values for the Al-Au-1, Al-Au-3, and Al-Au-4 samples. Prolonging the Au deposition time while simultaneously reducing the current density created a sluggish reactive environment conducive to the gradual growth of gold atoms. This resulted in the formation of distinct surface wrinkles on Al-Au-5, as illustrated in Figure 1e. On the contrary, when the plated current density was increased to 0.3 A/m^2^, Al-Au-6 showed the smoother surface, while the nodular diameter was larger. For the Al-Au-2 sample shown in Figure 1b, which featured a coincident current density and curtailed plating time of Au compared to Al-Au-1, the nodular morphology was retained. However, significantly smaller particles with diameters of approximately 0.5 µm were observed. These contributed to an enhanced level of compactness and uniformity in Al-Au-2. Therefore, the current density and deposition time of Au significantly impacted the surface morphology and structure.

**Figure 2:**
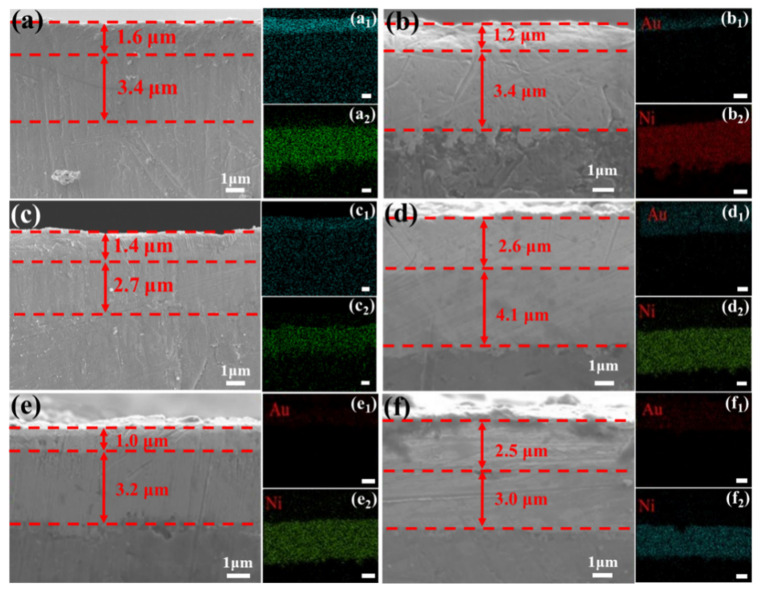
The cross-sectional morphologies and corresponding EDS mappings of (**a**–**a_2_**) Al-Au-1, (**b**–**b_2_**) Al-Au-2, (**c**–**c_2_**) Al-Au-3, (**d**–**d_2_**) Al-Au-4, (**e**–**e_2_**) Al-Au-5, and (**f**–**f_2_**) Al-Au-6. The scale bar is 1 μm.

The cross-sectional morphologies and corresponding elemental mappings of the Ni-Au coatings are shown in Figure 2. Evidently, the Ni-Au coating applied to the 2A12 aluminum alloy features a double-layer structure, comprising a Au layer and a Ni layer. The Ni-Au coating exhibited excellent interfacial strength, with no visible holes or defects at the interface between the Ni and Au layers, showcasing robust interface interaction. Generally, extending the Ni deposition time resulted in an increase in the thickness of the Ni layer. As illustrated in Figure 2a–c,e,f, when the plating time of Ni was maintained at 12 min, its thickness varied within a range of 2.7 to 3.4 μm. Prolonging the Ni deposition time to 20 min led to a significant thickness increase to 4.1 μm for Al-Au-4 (Figure 2d). Among the samples with the same Ni deposition time, Al-Au-3 displayed a slight decrease in the Ni layer thickness, which could be attributed to a reduction in the number of zincating cycles.

For the Au layer, when the deposition current density remains unchanged, its thickness also increases with the deposition time. As shown in Figure 2a–d, compared with other samples that had a Au-plated current density of 0.2 A/m^2^, the thickness of the Au layer in Al-Au-2 decreased significantly because of the short deposition time. Notably, Al-Au-4 showed an abnormal enlargement of the Au layer, which could be attributed to the influence of a longer Ni deposit. For the Al-Au-5 and Al-Au-6 samples, even though the charge deposited per unit area of the Au layer remained constant, Al-Au-5 exhibited a notable decrease in the thickness of the Au layer, whereas a significant increase was observed in Al-Au-6. This indicated that the thickness of the Au layer was significantly affected by the deposition current density rather than electroplating time, with a higher current density and longer duration resulting in a thicker Au layer.

### 3.2. Structures and Adhesion Forces of Ni-Au Coatings

**Figure 3:**
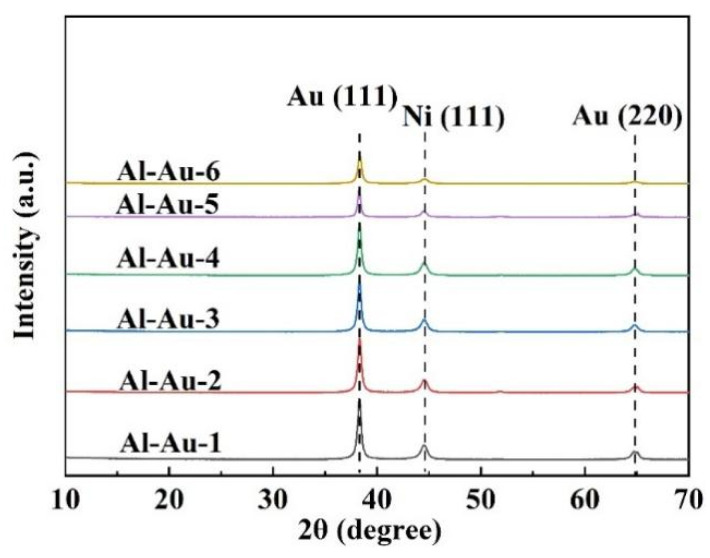
The XRD spectra of the Ni-Au coatings.

The crystal structures of samples were analyzed using XRD. In Figure 3, all samples exhibited characteristic peaks of Au and Ni [31]. Specifically, the peaks observed at 38.36°, 44.52°, and 65.10° correspond to the characteristic peaks for Au (111), Ni (111), and Au (220), respectively. Furthermore, the grain size of the Au layer was determined using the Scherrer formula, based on the Au (111) peak, as detailed below [32].

*D* = *Kλ*/(*β*cos*θ*) (2)

where *D* denotes the mean grain size in the direction perpendicular to the reflecting planes, *K* is the Scherrer constant, *λ* represents the wavelength of the incident X-ray, *θ* signifies the diffraction angle, and *β* denotes the full width at half maximum (FWHM) of the diffraction peak. The obtained *D* data are listed in Table 2. There were variations in the grain size information reflected by the XRD patterns among these samples.

Considering the influence of current density and electroplating time on grain size, it was evident that grain size was predominantly affected by deposition current density rather than deposition time. For samples Al-Au-1 to Al-Au-4, their grain sizes remained similar, ranging from 21.7 to 21.9 nm, despite the deposition time of the Al-Au-2 sample being halved. When the current density was diminished to 0.1 A/m^2^, the grain size of Al-Au-5 notably increased to 27.5 nm. By contrast, the Al-Au-6 sample exhibited the smaller grain size due to the higher deposition current density. A longitudinal comparison of the process parameters for Al-Au-1, Al-Au-5, and Al-Au-6 revealed that an increase in current density was associated with a decrease in the grain size of the samples [33].

**Figure 4:**
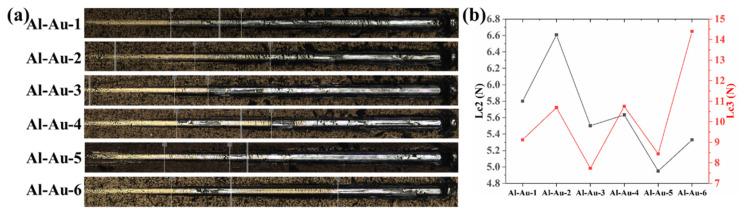
(**a**) Scratch morphologies and (**b**) adhesion strengths obtained for the Ni-Au coatings.

The adhesion strength of the Ni-Au coating was evaluated using the scratch method, with the results presented in Figure 4. During scratch testing, a load was applied to the coating and gradually increased from 0 to 20 N. The critical load (Lc), the normal force at the failure point, was mainly divided into three categories: Lc1, Lc2, and Lc3. Lc1 denotes the load at the beginning of the crack; Lc2 signifies the critical load corresponding to the initiation of coating debonding and is commonly employed to define the adhesion of the coating; and Lc3 represents the critical load associated with the complete peeling of the coating. In the process of scratching, as the indenter deformed the coating and substrate, a semi-circular crack emerged within the scratch trajectory after reaching Lc1. Following Lc2, a slight residue of the coating remained within the scratch, accompanied by conspicuous peeling on either side. Upon attaining Lc3, the coating experienced complete delamination and exhibited total failure.

In Figure 4a, the failure modes of all samples were consistent. Initially, upon applying the load, the Au layer underwent substantial plastic deformation, exhibiting a bright yellow color. As the applied load increased to 20 N, a partially silver metallic luster gradually emerged until the coating visibly began to spall. The adhesion strengths obtained are listed in Figure 4b. Notably, the Lc2 and Lc3 values of the Ni-Au coating displayed contrasting trends. For Lc2, Al-Au-5 exhibited the lowest value compared to other samples. Similarly, Al-Au-6 showed a relatively low Lc2 value as well. In contrast, Al-Au-2 displayed a higher Lc2 value when compared to Al-Au-1, Al-Au-3, and Al-Au-4. The relatively low adhesion strength in the Al-Au-3 samples could be attributed to fewer zincating cycles, leading to reduced bonding strength between the Ni layer and the Al substrate. This observation was also reflected in the Lc3 curve, emphasizing the critical importance of the zincating process for enhancing adhesion. In addition to the abnormal enlargement in Al-Au-6, both Al-Au-2 and Al-Au-4 showed relatively higher Lc3 values, implying their greater adhesion strength on the Al alloy. Considering the values of Lc2 and Lc3 comprehensively, it was evident that Al-Au-2 demonstrated both higher binding strengths, while Al-Au-3 showed the weakest performance. This suggested that optimizing the process parameters by increasing the number of Zn immersion cycles and simultaneously reducing the Au electrodeposition duration led to a more cohesive union between the three layers, thereby enhancing the bonding strength.

### 3.3. Anti-Corrosion Performance

**Figure 5:**
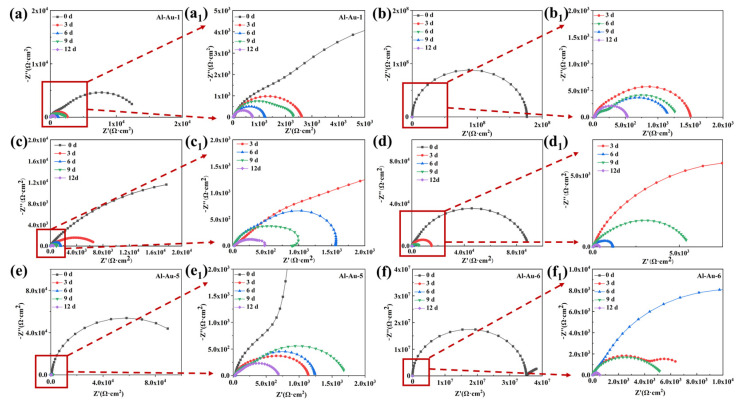
Nyquist plots of (**a**,**a_1_**) Al-Au-1, (**b**,**b_1_**) Al-Au-2, (**c**,**c_1_**) Al-Au-3, (**d**,**d_1_**) Al-Au-4, (**e**,**e_1_**) Al-Au-5, and (**f**,**f_1_**) Al-Au-6 in 3.5 wt.% NaCl solution.

In Figure 5, the corrosion resistance of Ni-Au coating was evaluated by electrochemical impedance spectroscopy (EIS) in 3.5 wt.% NaCl solution. For these samples, the diameter of the arc decreased continuously with increasing immersion time in NaCl solution, indicating that the continuous invasion of corrosive media had caused damage to the coating [34]. Samples Al-Au-1 and Al-Au-3 showed a relatively small capacitance arc diameter in the initial immersion. As the soaking time prolongs, the capacitance arc diameter of the sample decreased significantly. An abnormal increase in the capacitance arc diameter was observed at the 6–9 d immersion, possibly due to the corrosion medium penetrating into the interface between the Ni-Au coating and the Al substrate. This temporary hindering effect of corrosion products raised the capacitance arc diameter [35]. These observations demonstrated the poor anti-corrosion performance, which could be attributed to the poor surface condition and adhesion strength.

For Al-Au-5 in Figure 5e, the decrease in current density only resulted in an increased capacitance arc diameter when immersed for 0 d, which was an order of magnitude higher than that of Al-Au-1. During subsequent immersion periods, the Al-Au-5 samples showed an equivalent or worse impedance value. In contrast, Al-Au-6 revealed significantly higher impedance before the 9 d immersion, as shown in Figure 5f. It was manifest from the deposition parameters that the electrical charge of the Au layer in the Al-Au-1, Al-Au-5, and Al-Au-6 samples remained identical throughout the deposition process. Nevertheless, the Al-Au-6 sample exhibited the highest anti-corrosion properties, while the Al-Au-5 sample showed the lowest. This suggested that the deposition current density of the Au layer significantly influenced the protection performance of the Ni-Au coating, with higher current densities resulting in superior protection, rather than being determined by the total electricity used in the deposition process.

**Figure 6:**
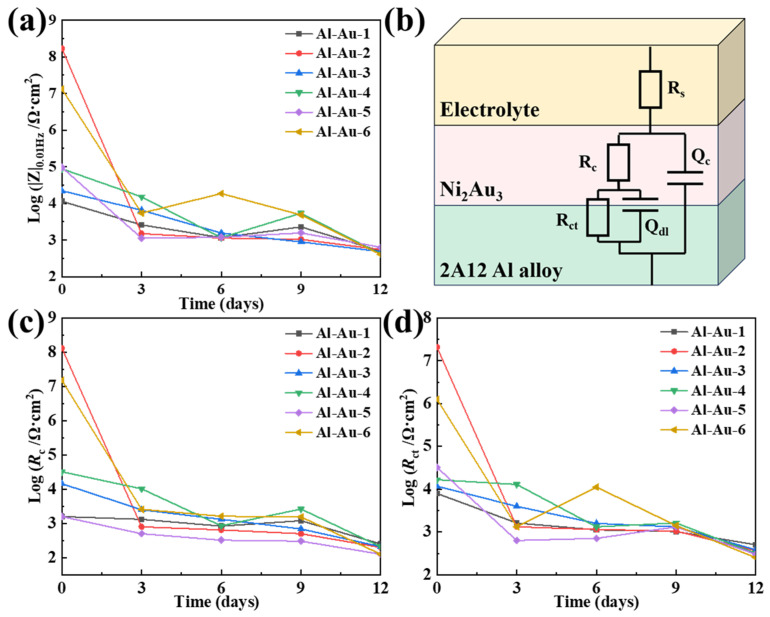
The variations in (**a**) log |Z|_0.01Hz_, (**c**) log *R*_c_, and (**d**) log *R*_ct_ of the Ni-Au coatings, and corresponding (**b**) equivalent electrical circuit.

Unlike the increased low-frequency impedance (|Z|_0.01Hz_) values observed in the aforementioned samples, Al-Au-2 displayed a gradual decline throughout the soaking period. Additionally, significantly high |Z|_0.01Hz_ values (1.73 × 10^8^ Ω·cm^2^) were noted initially, followed by a sharp decline to 1.58 × 10^3^ Ω·cm^2^ after 3 days of immersion, as depicted in Figure 6a. The excellent anti-corrosion performance indicated that the dense and uniform surface morphology of the Au layer served as the primary defense against the penetration of corrosive media. In the case of Al-Au-4 with an extended Ni deposition duration, the capacitance arc diameter was significantly increased by 1–2 orders of magnitude in the initial 0–3 d compared with that of Al-Au-1. The samples then revealed the same variation in trend and magnitude of impedance after 6 d immersion. This was attributed to the extended Ni deposition time, which ensured an adequate duration for the deposition of Ni atoms. The resulting thick and dense Ni layer served as a secondary protective barrier against corrosion.

The variation in EIS fitting parameters over immersion time is illustrated in Figure 6c,d, which were fitted using the equivalent electrical circuits (EECs) depicted in Figure 6b. Here, *R*_s_ denotes the resistance of the electrolyte solution [36]. *R_c_* and *Q_c_* correspond to a pair of components of the Ni-Au coatings, where *R_c_* represents the coating resistance and *Q_c_* is a constant-phase element connected in parallel with *R_c_* [37]. *R*_ct_ and *Q*_dl_ are used to describe the corrosion process. Specifically, *R*_ct_ is the charge transfer resistance between Ni-Au coating and substrate, and *Q*_dl_ denotes the electric double-layer capacitance. As soaking time progresses, resistance diminished markedly, with temporary rises at specific intervals due to the barrier effect of corrosion product formation.

It was obvious that the *R*_c_ and *R*_ct_ values of Al-Au-2 and Al-Au-6 were higher than those of the others in the initial immersion. And then they declined sharply after immersion for 3 d. This indicated that the smooth surface condition and dense Au layer could greatly improve the corrosion resistance of aluminum alloy in the short term. Once the corrosive medium penetrated into the interface of the Ni-Au coating/Al substrate, corrosion proceeded quickly due to the significantly higher potential of the Au layer. The Al-Au-1 and Al-Au-5 samples exhibited relatively smaller *R*_c_ and *R*_ct_ values both before and after immersion compared to the Al-Au-2 and Al-Au-4 samples. This was primarily due to the presence of defective surface structures that were susceptible to corrosive media invasion, thereby resulting in diminished corrosion resistance. During the whole immersion process of 12 d, Al-Au-4 showed relatively higher *R*_c_ and *R*_ct_ values. This was ascribed to the dense Au layer and the thick Ni layer. The former constituted the initial protective barrier, ensuring superior anti-corrosion performance. And the latter caused by the extended Ni deposition time in Al-Au-4, which furnished an additional protective layer, further safeguarding against the incursion of corrosive media.

**Figure 7:**
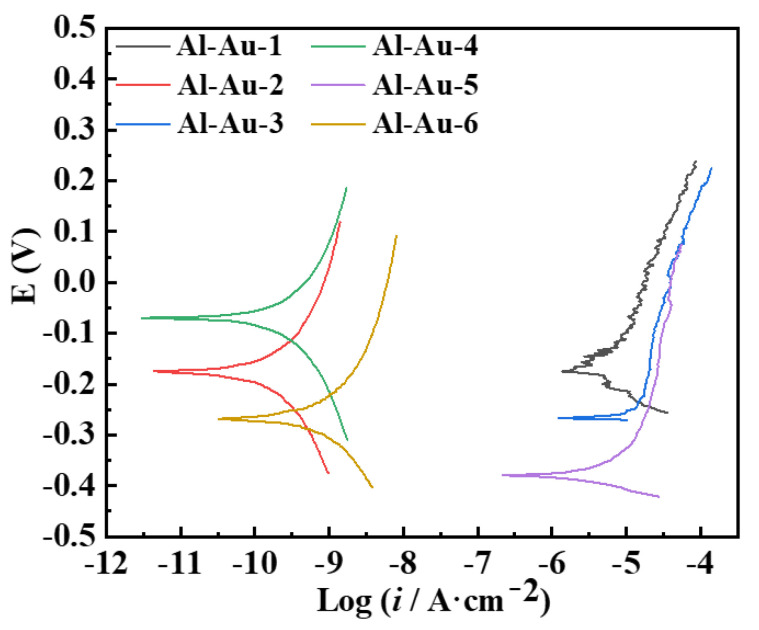
Polarization curves of the Ni-Au coatings.

Additionally, the corrosion resistance behavior of different Ni-Au coatings was further evaluated using potentiodynamic polarization curves, and the results are shown in Figure 7. Corrosion current density (*i*_corr_) is generally recognized as a crucial indicator for assessing the corrosion rate of metals. A higher *i*_corr_ correlates with a faster corrosion rate, thereby indicating poorer anti-corrosion performance. It was evident that Al-Au-2, Al-Au-4, and Al-Au-6 exhibited lower *i*_corr_ values compared to Al-Au-1, Al-Au-3, and Al-Au-5, indicating significantly superior corrosion resistance performance. Comparing the relevant process parameters of Al-Au-1 and Al-Au-4, it could be confirmed that extending the Ni-plated time could greatly improve the corrosion resistance of the Ni-Au coating. When the thickness of the Ni layer was comparable, the density and thickness of the Au layer played a crucial role in determining the protective performance of the coating. In the case of thicker Au layers, the presence of coating defects led to a marked deterioration in the protection performance. This was primarily due to the higher self-corrosion potential of Au, which caused the defect site to exhibit a large cathode-to-anode ratio, thereby accelerating the corrosion process. This was consistent with the results of EIS. Among Al-Au-2, Al-Au-4, and Al-Au-6, Al-Au-2 showed lowest *i*_corr_ value. Hence, despite Al-Au-4 exhibiting a higher self-corrosion potential (*E*_corr_) compared to Al-Au-2, Al-Au-2 still demonstrated better corrosion protection performance. This suggested that extending the deposition time of Au did not necessarily enhance performance when other parameters were held constant. Instead, the surface state and density of the coating exerted a more significant influence on its protective efficacy.

**Figure 8:**
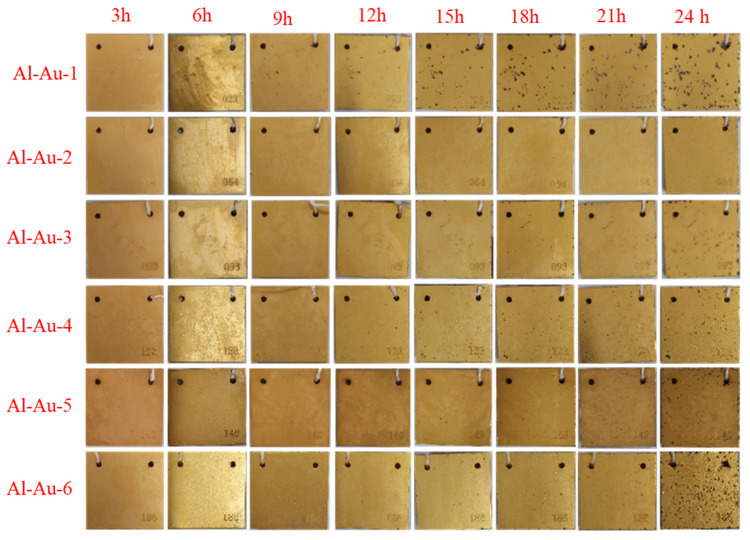
Optical morphologies of Ni-Au samples after salt spray experiments at different times.

The optical images of salt spray tests with different Ni-Au coatings at different usage times are shown in Figure 8. All samples initially provided effective protection upon exposure. However, as the duration of the salt spray test progressed, the defect area on these samples gradually expanded. Notably, Al-Au-1 exhibited the poorest corrosion resistance, characterized by the fastest rate of pitting corrosion development. Conversely, the other samples exhibited superior protective properties to varying degrees during the salt spray test. To accurately evaluate the protective performance of the Ni-Au coating, its anti-corrosion grade (*R*_P_) was classified according to the standard [38], as shown in Table 3. Obviously, Al-Au-2 showed the best protective performance during the whole stages of salt spray testing, and its pitting behavior increased slowly over time. For Al-Au-3 to Al-Au-6, there was minimal pitting on the surface with a *R*_P_ between 8 and 9 prior to 21 h of exposure, after which the surface condition deteriorated rapidly by the 24 h mark. In summary, based on the optical images from the salt spray experiment, it could be preliminarily concluded that Al-Au-2 exhibited superior corrosion resistance compared to the other samples, while Al-Au-1 showed the poorest. This observation aligned with the results of the electrochemical tests, and could be attributed to the thick Ni layer, dense Au layer, and smooth surface microstructure in Al-Au-2.

**Figure 9:**
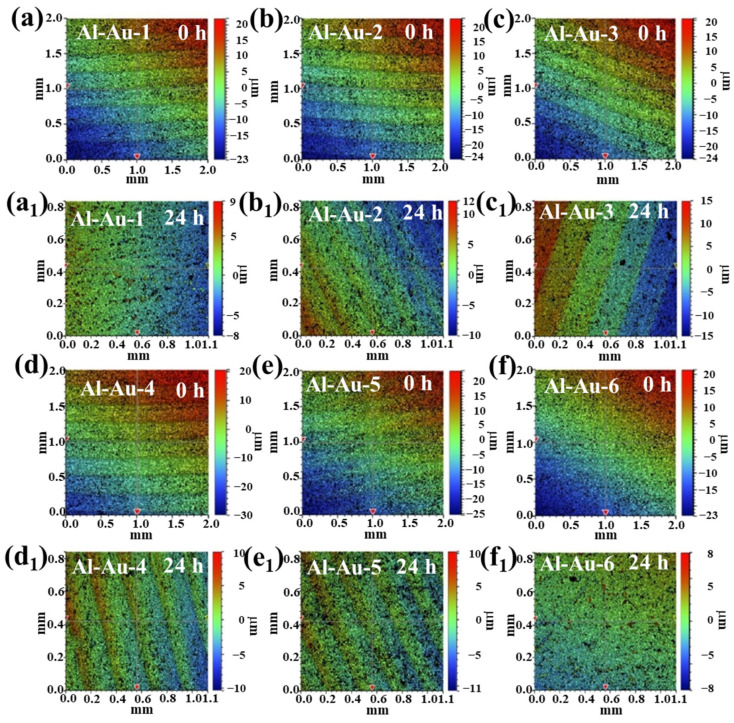
Three-dimensional white light interference surface morphologies of the Ni-Au coatings (**a**–**f**) before and (**a_1_**–**f_1_**) after the salt spray test for 24 h.

The three-dimensional morphologies of Ni-Au coatings on 2A12 aluminum alloys after 24 h salt spray were evaluated using a white light interferometer, and the results are shown in Figure 9. Different colors signified variations in the surface height of the sample, influenced by discrepancies in the thickness of the aluminum alloy. Periodic striations were a consequence of aluminum alloy surface machining. Figure 9a–f depict the surface conditions of Ni-Au coatings before the salt spray tests. Small black spots on the surface might be attributed to microscopic irregularities on the specimen surface. All specimens exhibited a nearly identical surface state. After 24 h of salt spray exposure, large black spots became evident on the surfaces of the samples, as illustrated in Figure 9a_1_–f_1_. Notably, the surface of Al-Au-2 maintained the best condition after 24 h of corrosion, which was consistent with the optical photograph results from the salt spray test.

**Figure 10:**
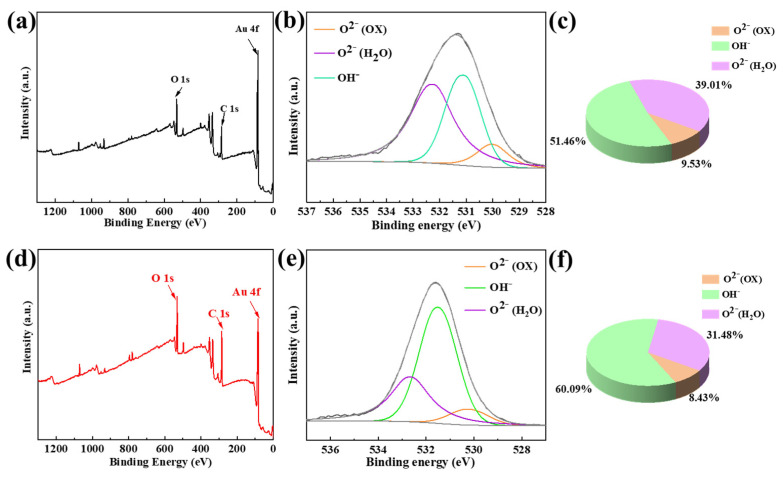
Results showing the (**a**,**d**) overall XPS survey, (**b**,**e**) O 1s high-resolution spectrum of Al-Au-5, and (**c**,**f**) corresponding content ratio of the O-containing groups before and after the salt spray test.

Through optical photography and salt spray three-dimensional morphology analysis, it was determined that Al-Au-5 exhibited relatively poor anti-corrosion performance. To investigate the chemical composition of corrosion products, XPS analysis was conducted on Al-Au-5 before and after salt spray exposure, and the results are shown in Figure 10. The overall XPS spectrum of Al-Au-5 remained consistent before and after the salt spray test, displaying comparable peaks for O 1s (529.0 eV), C 1s (284.8 eV), and Au 4f (84.0 eV). The presence of C element might be due to the introduction of external environmental pollution or residual organic matter during the electroplating process. No photoelectric signals for the Ni and Zn elements were detected either before or after the salt spray exposure. This indicated that there was no distinct delamination or corrosion of the Ni layer and Zn in the coating, primarily due to the corrosion of the Al substrate after defects appeared in the coating. From the high-resolution O spectrum, distinct peaks were identified corresponding to O^2−^, OH^−^, and adsorbed H_2_O at 530.5 eV, 531.7 eV, and 532.7 eV, respectively [39]. Among these, O^2−^ and OH^−^ mainly corresponded to the corrosion products Al_2_O_3_ and Al(OH)_3_ formed in Al-Au-5, as illustrated in Figure 10c,f. Notably, the OH^−^ content following the corrosion of Al-Au-5 was higher than that prior to corrosion. This meant that the corrosive medium penetrated into the Al alloy substrate through the defective Ni-Au coating, leading to the formation of hydroxides.

### 3.4. Life Prediction of Al-Au-2

The grey model method is a purely mathematical approach to predict material life based on grey correlation analysis. The core concept involves fully digitizing all performance indicators that reflect the lifespan of materials. It requires identifying patterns from a limited amount of data to calculate unknown data that have been in the grey area for an extended period. This approach demonstrates a certain level of accuracy in predicting the service life of materials. The specific prediction model is shown as Equation (1). Among all the samples, Al-Au-2 exhibited the highest corrosion resistance, making it a favorable candidate for lifespan evaluation. To enhance the accuracy of the prediction, the electrochemical parameter *R*_c_ was supplemented for Al-Au-2 from 0 d to 9 d prior to corrosion, as presented in Table 4.

To develop a mathematical model for the electrochemical parameter *R*_c_ values, assuming *x*^(0)^(*t*) = {8.15, 2.90, 2.81, 2.30}, the data were accumulated to obtain *x*^(1)^(*t*) = {8.15, 11.05, 13.86, 16.16}. For {*x*^(1)^(*t*)} governed by an exponential law, let Y^(1)^ represent the set of equally spaced subsequences derived from {*x*^(1)^(*t*)}. Subsequently, the grey differential equation (Equation (3)) on Y^(1)^ can be expressed as

*x*^(0)^(*t*) + *ay*^(1)^(*t*) = *u* (3)

The shadow equation (Equation (4)) is derived from the equation above as

(4) $$\frac{dx^{\left(1\right)}}{dt}+ax^{\left(1\right)}=u$$

where *a* is the development coefficient and *u* is the grey action. The parameters *a* and *u* were determined using the least squares method [*a*, *u*]^T^ = (C^T^C)^−1^ C^T^ Z_N_^T^ according the following equations:

$$C=\left[\begin{array}{cc} -[x_{1}^{\left(1\right)}(2)+x_{1}^{\left(1\right)}(1)]/2 & 1\\ -[x_{1}^{\left(1\right)}(3)+x_{1}^{\left(1\right)}(2)]/2 & 1\\ \ldots & 1 \end{array}\right]$$

*Z*_N_ = [*x*^(0)^(2), *x*^(0)^(3), …]

The specific calculation process is shown in Equations (5)–(7):

(5) $$C=\left[\begin{array}{cc} -9.60 & 1\\ -12.455 & 1\\ -15.01 & 1 \end{array}\right]$$

(6) $$Z_{N}={\left[2.9,\;2.81,\;2.3\right]}^{T}$$

[*a*, *u*]^T^ = (C^T^C)^−1^C^T^Z_N_ = [0.18, 5.01]^T^ (7)

Hence, the model coefficient A = $\left[x^{\left(0\right)}(1)\;-\frac{u}{a}\right]$ = −19.7. The resistance of the coating, assessed as being on the verge of failure after corrosion, was 100. Consequently, lg*R*_c_ was taken as 2 for the subsequent analysis. Specifically, the salt spray service life of Al-Au-2 can be calculated by Equation (8):

(8) $$t=\frac{3\times ln(\frac{-19.7\times (1-e^{-0.18})}{2})}{0.18}=12.89\;(d)$$

From the electrochemical data in Figure 6c, it was evident that the *R*_c_ values sharply decline after 3 days of immersion, followed by a gradual decrease. Upon immersion for 12 days, log*R*_c_ reached 2.3, closely matching the actual calculated result. According to the life prediction analysis, the coating resistance was projected to drop to 100 after approximately 12.89 days of immersion.

### 3.5. Corrosion Protection and Failure Mechanisms

The Au layer on the surface provides excellent conductivity, and its inherent inert characteristics along with a high electrode potential (1.68 V) ensure its exceptional chemical stability and corrosion resistance. To further enhance the adhesion strength of the Au layer with the Al alloy substrate, a Ni layer is utilized as the pretreatment layer. For the anti-corrosion protection coating, the potential difference between the Au layer and the intermediate Ni layer is minimal (△V < 0.05 V), allowing the corrosion tendency to be disregarded. Consequently, when the coating remains dense and intact, it exhibits superior corrosion protection properties.

However, based on the surface conditions of the Ni-Au samples after salt spray testing, the corrosion failure mode is identified as pitting corrosion, consistent with what is observed in pure Al alloys. The protective properties of the samples vary due to the influence of differing process parameters on the coating structure, including coating thickness, surface condition, and adhesion strength. This variation is attributed to the fact that the potential of the Ni-Au layer remains higher than that of the Al alloy, particularly for the 2A12 alloy, which contains intermetallic compounds (Al_2_CuMg, *S* phase, and Al_2_Cu, *θ* phase), making it susceptible to dealloying corrosion [40,41]. When the Ni-Au coating on the surface of the 2A12 aluminum alloy is defective (such as holes) or damaged, an activation–passivation corrosion cell forms. The Ni-Au plated layer acts as the cathode and has a significantly larger area compared to the activated area at the site of film damage, leading to the phenomenon of a large cathode–small anode. This configuration facilitates deeper corrosion that eventually forms corrosion pits [36,39].

In fact, regardless of the material used as the cathode in the corrosion galvanic cell, such as a rich copper region, a Ni layer, a Au layer, or C introduced during electroplating, the corrosion reaction of the aluminum alloy in neutral NaCl solution can be expressed by the following equation:

(9) $$\mathrm{Al}\;\to 3e^{-}+{\mathrm{Al}}^{3+}$$

(10) $$O_{2}+2H_{2}O+4e^{-}\to 4{\mathrm{OH}}^{-}$$

Various cathodes manifest distinct electrochemical sequences, giving rise to dissimilar potential differences when coupled with the anode. The larger the potential difference, the faster the corrosion rate. The incorporation of Ni and Au, which possesses higher self-corrosion potentials, resulted in a greater potential difference with Al and Mg, thereby generating the thermodynamic driving force for galvanic corrosion. Therefore, for metal anti-corrosion coatings, it is crucial to choose materials with similar potentials within the galvanic series to mitigate the risk of substantial galvanic corrosion driving forces arising from significant potential differences.

Nevertheless, the direct contact with dissimilar metals is often difficult to avoid. Hence, it is crucial to minimize porosity of metal anti-corrosion coatings, suppress the adsorption of active ions (such as Cl^−^) in the corrosion sensitive area, and prevent the occurrence of the large cathode–small anode configuration. Moreover, the augmentation of the Ni intermediate layer thickness within the coating can prevent pinholes and other coating defects from reaching the base of the aluminum alloy substrate, effectively thwarting the corrosion of the substrate. Furthermore, immersing the coating with protective agents, such as organic or inorganic corrosion inhibitors, coating precursor solutions, and so on, can also isolate the contact between corrosive media and areas susceptible to pitting, thereby enhancing the corrosion resistance of the Ni-Au coating [40,41].

## 4. Conclusions

In this study, various Ni-Au coatings were designed through the modification of the process parameters. The impact of surface morphology, crystal structure, and thickness of coatings on corrosion resistance was experimentally investigated. Furthermore, a comprehensive analysis of their anti-corrosion mechanisms was conducted, leading to the following conclusions:

(a) The higher the current density and the longer the deposition time, the thicker the Au layer became. However, it was not necessarily true that a thicker Au layer equates to better protective performance. Compared with the thickness of the Au layer, the grain size of the Au layer had a more significant effect on the surface state and density of the Au layer. Smaller grain size resulted in a smoother surface and better protection performance.
(b) Increasing the deposition current density of the Au layer significantly improved the surface condition of the coating, rather than being determined by the total electricity used in the deposition process. Additionally, extending the Ni deposition time could effectively increase the thickness of the Ni layer, providing a secondary protective barrier against corrosion. Zinc immersion subtly affected the bonding strength of the coating, significantly influencing its resistance to the incursion of corrosive media.
(c) The surface state and density of the coating exert a more noticeable effect on its protective efficacy. Al-Au-2 exhibited superior corrosion resistance compared to the other samples, which was attributed to the thick Ni layer, dense Au layer, and smooth surface microstructure in Al-Au-2.
(d) The *R*_c_ value obtained from the electrochemical fitting of samples was utilized to develop a corrosion life prediction model based on grey theory. Applying this model to Al-Au-2, a life prediction analysis was conducted. The results indicated that the protective properties of Al-Au-2 sample failed after 12.89 days of immersion with *R*_c_ reaching 100 Ω·cm^2^. This aligns with the trends observed in the electrochemical experiments.

This study deepens the understanding of the interplay between process parameters and the microstructure of the Ni-Au coating, establishing a theoretical foundation for evaluating reliability and service life in aggressive corrosive environments. Building on these findings, future research will further investigate the synergistic effects of multiple parameters and establish multi-environmental factor experiments to accurately simulate real-world usage conditions.

## Figures and Tables

**Table 1:** Process parameters for Ni-Au coatings on 2A12 aluminum alloy.

**Number**	**Zincating Times**	**Ni Plating Time** **(min)**	**Au Electroplating Current Density** **(A/m^2^)**	**Au Electroplating Time** **(min)**
Al-Au-1	2	12	0.2	60
Al-Au-2	2	12	0.2	30
Al-Au-3	1	12	0.2	60
Al-Au-4	2	20	0.2	60
Al-Au-5	2	12	0.1	120
Al-Au-6	2	12	0.3	40

**Table 2:** The Au deposition parameters and corresponding grain sizes of the Ni-Au coatings.

Number	Au-Plated Current Density (A/m^2^)	Au-Plated Time (min)	Grain Size of Au (nm)
Al-Au-1	0.2	60	21.7
Al-Au-2	0.2	30	21.9
Al-Au-3	0.2	60	21.9
Al-Au-4	0.2	60	21.7
Al-Au-5	0.1	120	27.5
Al-Au-6	0.3	40	20.8

**Table 3:** The *R*_P_ of Ni-Au coatings on 2A12 aluminum alloy after salt spray experiments at different times.

Time (h)	Al-Au-1	Al-Au-2	Al-Au-3	Al-Au-4	Al-Au-5	Al-Au-6
3	9	10	10	9	10	9
6	8	10	9	9	9	9
9	8	9	9	9	9	9
12	8	9	9	9	9	9
15	6	9	9	8	9	9
18	6	9	9	8	9	9
21	6	9	8	8	8	8
24	6	8	7	7	6	6

**Table 4:** Electrochemical parameters of Al-Au-2.

Time/d	lg*R*_c_
0	8.15
3	2.90
6	2.81
9	2.30

## Data Availability

The original contributions presented in the study are included in the article. Data will be made available on request.
